# Delta-Like Ligand (DLL)1 Expression in Early Mouse Decidua and Its Localization to Uterine Natural Killer Cells

**DOI:** 10.1371/journal.pone.0052037

**Published:** 2012-12-28

**Authors:** Karina Y. Degaki, Zhilin Chen, Aureo T. Yamada, B. Anne Croy

**Affiliations:** 1 Department of Biomedical and Molecular Sciences, Queen’s University, Kingston, Ontario, Canada; 2 Institute for Biology, University of Campinas (UNICAMP), Campinas, São Paulo, Brazil; Konkuk University, Republic of Korea

## Abstract

Uterine vascular changes, critical for pregnancy success, occur at each implant site during endometrial decidualization. Mesometrial decidualization recruits high numbers of angiogenic, uterine Natural Killer (uNK) cells that trigger midpregnancy spiral arterial remodeling. We postulated that uNK cells contribute to early decidual angiogenesis as endothelial-cell extrinsic sources of Delta-like ligand 1 (DLL1), a molecule that induces endothelial tip cell differentiation and orthogonal vascular growth in other tissues. Virgin uteri expressed *Dll1* mesometrially and anti-mesometrially and relative expression increased in both anatomic regions as pregnancy progressed. Analyses of transcripts from gd10.5 uNK cells flow sorted on the basis of expression of *Dolichos biflorus* agglutinin (DBA) lectin revealed that DBA+ but not DBA- uNK cells expressed *Dll1*. Immunostaining at gd4.5 found DLL1-expressing cells rare. At gd6.5, DBA+ uNK cells at all stages of maturation expressed DLL1. By gd10.5, DLL1 immunoreactivity was strongly expressed by some but not all DBA+ uNK cells and more weakly by DBA- cells. DLL1+ cells were mesometrially stratified and concentrated within central decidua basalis. Our data suggest that uNK cells have the potential to induce endothelial tip cell differentiation and to promote non-planar vascular growth within early decidua basalis.

## Introduction

The mammalian Notch signaling pathway is composed of four receptors (Notch 1–4) and five ligands (Jagged1 and 2 and Delta-like ligand (DLL) 1, 3 and 4). Notch uses cell contact-dependent signaling mechanisms triggered by the binding of Notch ligands to their receptors [1]. Signal transmission generally occurs between neighboring cells that express high levels of either the receptor or the ligand, although receptor-ligand co-expression occurs in some cell types, such as endothelium [2]. Notch activity in endothelial cells is essential for mouse development and successful pregnancy with the receptors Notch1 and Notch4 and the ligands Jagged1, DLL1 and DLL4 having major expression [2], [3]. Notch1 and Notch4 have similar roles promoting arterial cell-fate selection and angiogenesis [4]. Jagged1 plays roles in vascular smooth muscle development essential for postnatal survival [5], [6]. Loss of one copy of *Dll4* is a midpregnancy, embryonic lethal due to arterial-venous malformations [7] while heterozygosity at *Dll1* results in a microscopic vascular anomaly compatible with life [1]. Studies of neonatal retinal vessels using *Dll1* heterozygotes identified reduced endothelial tip cell development and impaired vascular branching in deeper layers compared with *+/+* controls. That is, orthogonal branching was more impaired than planar branching. Of importance, DLL1 was shown to be a product of extravascular cells [1].

Another tissue with rapid centrifugal advancement of a vascular plexus is the mouse implantation site [8]. Shortly after hatched blastocyst implantation on the anti-mesometrial (AM) side of the uterus, late on gestation day (gd)3, decidualization of the uterine stroma begins. Maternal neoangiogenesis commences after about 24 h later at gd5.0 [9] radiating from the embryonic crypt [8], [10]. Successful decidual development, including decidual microvascular development, is essential for completion of implantation in mice [11]–[13], humans [14], [15] and other species with hemochorial placentation [16], [17]. Steroid hormone regulation of Notch signaling pathways is implicated in uterine decidualization and decidual vascular development in mice, human and baboons [18], [19]. Roles for the Notch ligands have received less attention. [20]–[22].

Onset of decidualization in mice is followed rapidly by a massive influx of extravascular and intravascular Natural Killer lymphocytes (uNK cells) into each implant site [23], [24]. UNK cells and other leukocyte subtypes localize to the mesometrial (M) decidua basalis and would be present in the decidual samples in which Notch signaling has been demonstrated. At gd6.5, 50% of uNK cells express the surface lectin *Dolichos biflorus* agglutinin (DBA). The proportion of this uNK cell phenotype increases to 90% by gd10.5 when the effects of uNK cells on the promotion of structural changes in spiral arteries are quantifiable [25]. From adoptive cell transfer studies, DBA+ uNK cells were shown to be a subset that homes to the uterus from peripheral organs [25]. At midpregnancy (gd10.5), the DBA+ rather than DBA- subset contains the strongly angiogenic uNK cells [26]. The DBA+ uNK cell subset synthesizes vascular endothelial growth factor (VEGF) [27], [28], placenta growth factor (PGF) [29], ephrin B2 (EFNB2) [30], CD31 [8] and other molecules important to endothelial cells. UNK cells are found only at implant sites and not between them. At implant sites, uNK cells are found only mesometrially where they surround major branches from the uterine artery that supply each placenta. Both whole mount *in situ* immunohistochemistry and immunohistochemical staining of decidual tissue sections indicate that the highest density of new vessels in mouse implant sites is in the uNK cell-enriched, decidua basalis [8], [27]. The special angiogenic functions attributed to uterine but not to blood NK cells [26], [31] led us to postulate that uNK cells may be a source of endothelial cell-extrinsic DLL1 and that their recruitment to early decidua basalis would elevate numbers of endothelial tip cells to prepare a rich, 3-dimensional, vascular network to support the disc-shaped mouse placenta that completes its development in this region at approximately gd9.5–10. Molecular and immunohistochemical findings support this hypothesis.

## Materials and Methods

### Animals

Animal use was carried out in compliance with recommendations of the Canadian Council on Animal Care’s Guide to the Care and Use of Experimental Animals under protocols approved by University Animal Care Committee (UACC), Queen’s University. Inbred C57BL/6 (B6) and randombred CD1 mice were purchased from Charles River (St. Constant, QU). All mice were maintained with water and food “ad libitum” and 12 h light cycles. For pregnancies, 8-to 10-wk old females were caged overnight with syngeneic males, and vaginal plug detection was called gd0.5. Mice were euthanized by cervical dislocation and uteri were dissected and prepared distinctly for each protocol as described below. Vaginal smears and ovarian observation at post mortem were used to determine stage of estrus in virgin mice.

### Whole Mount *in situ* Immunohistochemistry

For whole mount staining, B6 uterine dissections were conducted under dissection microscope magnification as previously reported [8]. For gd4.5, uteri were bisected at the cervix and mid uterine horn then halved longitudinally to give M or AM tissue. For gd6.5 or 7.5, the AM uterine wall was incised, reflected mesometrially and trimmed to leave only a small tag of landmark myometrium. Decidual capsules were then halved midsagittally to open the embryonic crypt. Dissected tissues were then placed into 200 µL PBS supplemented with 1% bovine serum albumen (BSA) and 0.1% sodium azide (PBA) in a 5 mL tube on ice, 10 ug/mL of a blocking antibody to the IgG Fc receptor (anti-CD16/CD32 (supernatant of hybridoma 2.4G2, ATCC Bethesda MD)) were added and then CD45-FITC (1.5 µg/mL, 25-0451-81, eBioscience) and CD31-PE (0.8 µg/mL, 553373, BD Pharmingen) or DLL1-PE (0.8 µg, 128307, BioLegend, Cedar Lane Laboratories, Burlington ON, Canada) were added. After 1 h of incubation with rotation at 4°C, an excess of PBA was added to each tube and tissues were removed, placed on microscope slides, cover slipped, examined using epifluorescence microscopy and photographed.

### RNA Analysis

Virgin, gd4.5 and gd5.5 B6 uteri were dissected into M and AM tissue as described above for gd4.5 whole mounts. In separate dishes containing PBS, M or AM tissue was placed, lumen side up and small curved forceps were used to gently scrape the mucosa away from the uterine wall. At gd4.5 and 5.5, conceptus cells would be present predominantly in AM scrapings. At gd6.5, the decidual capsule containing the early primitive streak embryo can be dissected from the uterine wall. Dissected deciduas were opened under microscopic observation using #5 watchmaker’s forceps (Fine Science Tools, North Vancouver, B.C.), and the egg cylinder was removed. Then each decidual capsule was halved transversely into M and AM samples and similar samples from one pregnancy were pooled for RNA isolation. By gd10.5, AM decidua has regressed and was not studied while the M region has developed a second area distinct from decidua basalis. This transient region forms between the layers of uterine muscle only at implant sites, is enriched for dividing uNK cells [24] and is known as the mesometrial lymphoid aggregate of pregnancy (MLAp). To dissect gd10.5 uteri, the uterus was incised anti-mesometrially and the fetus and its membranes were discarded. The placenta was removed from the uterine wall, placed with the umbilical cord remnant down and the upper, whitish decidua basalis was removed. MLAps were harvested from the uterine wall with fine curved iridectomy scissors. For both tissues, all samples from a single litter were pooled. At least two females were independently prepared at each time point and their uterine samples were analyzed in multiple independent experiments.

Total RNA was prepared from M and AM virgin endometrium, gd4.5, gd5.5 and gd6.5 B6 M and AM decidua and from gd10.5 B6 decidua basilis and MLAp using Qiagen RNeasy Mini Kit. cDNA was synthesized from 0.5 µg total RNA using Invitrogen SuperScript® III First-Strand Synthesis System. The resulting cDNA was used as template for qRT-PCR. qRT-PCR used the ABI Prism 7500 system with TaqMan® Gene Expression Master Mix (Applied Biosystems, Foster City, CA). TaqMan® Gene Expression Assays containing *Hprt1*primer and TaqMan probe (Mm01545399_m1) were purchased from ABI. PrimeTime® qPCR Assays containing primers and TaqMan probes were purchased from Integrated DNA Technologies, Inc. for Mm.PT.51.8548995 (*Dll1*). PCR conditions were initial incubation (2 min; 50°C), enzyme heat activation (10 min; 95°C), 40 cycles (15 s at 95°C; 1min at 60°). Relative expression of target transcripts was normalized to hypoxanthine guanine phosphoribosyl transferase-1 (*Hprt1)* transcripts.

### Separation of DBA+ and DBA- uNK Cell Subsets and their Gene Expression Analysis by Quantitative qRT-PCR

Gd10.5 decidua basalis and MLAp were dissected as above, mechanically prepared as pooled cell suspensions and sorted by flow cytometry as previously described and validated [26]. Randombred CD1 mice that have large litters were used since neither adequate cell numbers nor RNA of sufficient quality was obtained after several gd10.5 B6 cell sorts. Two independent CD1 cell sorting experiments were conducted. Washed cells were incubated in 1% BSA, then stained (30 min, 4°C) with FITC-conjugated DBA (0.1 µg/mL; Sigma-Aldrich) and antibodies from eBioscience (San Diego, CA, USA): PE-conjugated anti-mouse CD122 (1/100; clone 5H4;) and PE-Cy5-conjugated anti-mouse CD3 (1/100; clone 145-2C11). Forward and side scatter properties were used to set the initial gates, then CD3-CD122+ cells were gated, sorted as DBA- or DBA+ cells and collected using an EPICS Altra Flow Hy-PerSort Cytometer (Beckman Coulter, Mississauga ON). Sorted cells were lysed and RNA was isolated using a PicoPure isolation kit (Molecular Devices; Toronto, ON) following manufacturer’s instructions. RNA was reverse transcribed and amplified using the Ovation Pico WTA System (NuGEN, San Carlos, CA) to PCR template cDNA.

### Immunohistochemistry for Detection of DLL1 and DBA Lectin Reactive Cells

Six-micrometer cryostat sections were cut from O.C.T.-embedded gd6.5 and gd10.5 B6 and CD1 implant sites, mounted onto coated slides (Superfrost Plus, Fisher Scientific, Toronto ON) and fixed (100% acetone, 15 min, 4°C). Sections were blocked (1% BSA, 30 min, 20°C), before overnight incubation (4°C) with anti-DLL1-PE (0.8 µg/mL, 128307, BioLegend). Sections were washed (PBS), incubated (1 h, 20°C) with FITC-DBA lectin (2 µg/mL, Sigma, Oakville, ON, Canada) then cover slipped with 4′,6-diamidino-2-phenylindole (DAPI) supplemented mounting medium (DAPI Gold with Anti-Fade Agent, Molecular Probes; Burlington, ON, Canada). Sections were photographed under epifluorescence with reference alignment using Zeiss Axiomat and Axiovision image analysis software (Zeiss; Toronto, ON, Canada). Archived, gd10.5 B6 paraffin embedded tissue sections co-stained for DBA lectin and periodic Acid Schiff’s reagent (PAS ) [25], a reagent that recognizes all granulated uNK cells, were studied microscopically for orientation and photographed.

### Statistical Analyses

Data are expressed as means±SEM. Statistical analyses were performed using Prism 4 software (GraphPad Software, Inc.). Statistical significance of the difference between two sets of data was assessed by one away ANOVA with Tukey’s post test. P<0.05 was considered significant.

## Results

### Mesometrial Decidual Vessels Differ to Vessels in Anti-mesometrial Decidua

**Figure 1:**
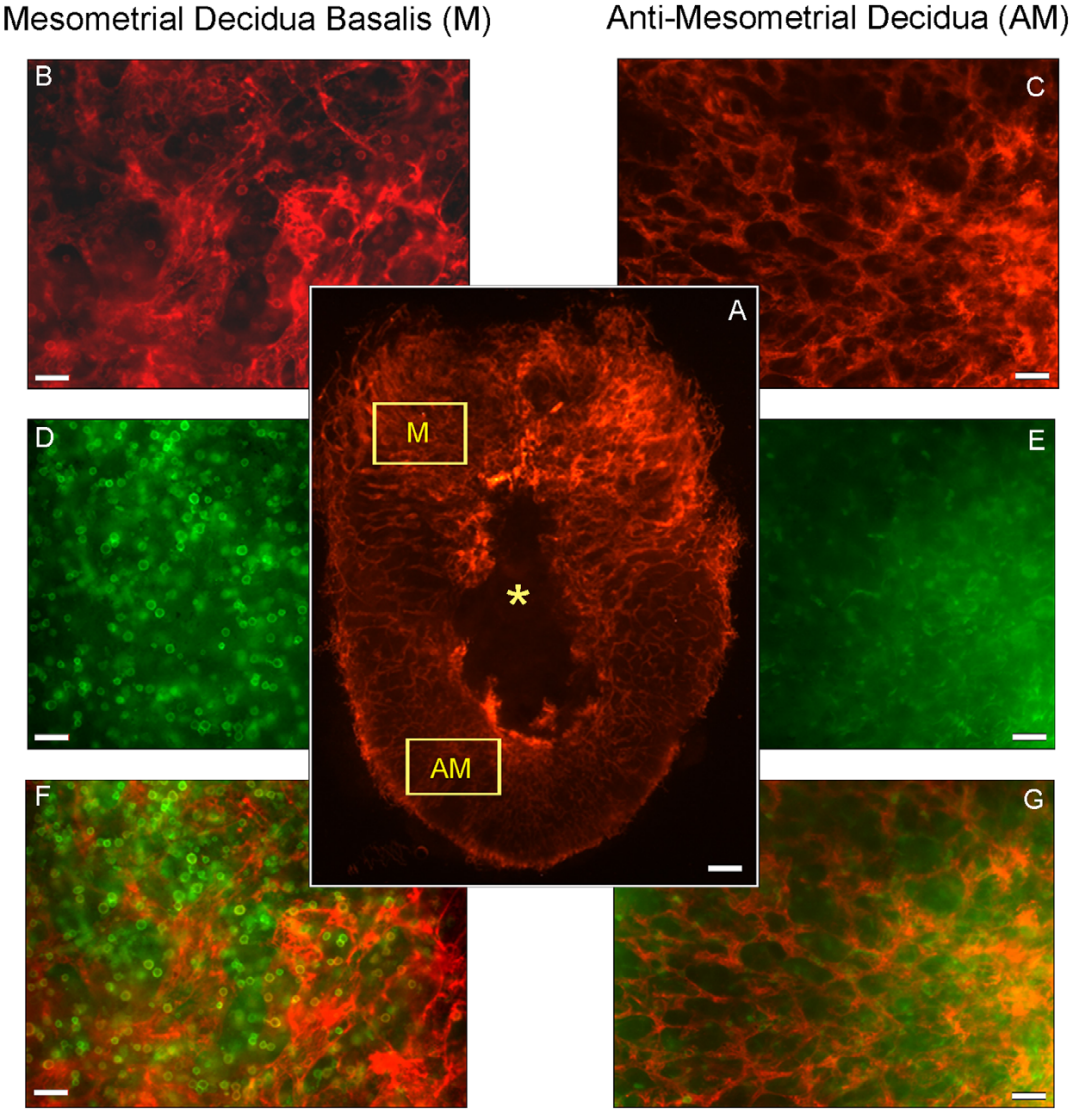
Whole mount in situ immunohistochemistry of gd7.5 B6 implantation sites co-stained with PE-CD31 (red) and FITC-CD45 (green). Low power image (A) is a for orientation. The mesometrial side of the uterus is uppermost; the central black region (*) represents the embryonic crypt. More CD31 expression is present mesometrially than anti-mesometrially or in lateral (L) decidua. Images B, D, F are of the same field (represented by the boxed M) of the mesometrial uterus. Images C, E, G are of the same field (represented by the boxed AM) from the anti-mesometrial uterus. Image (B) illustrates the larger, web-like, less linked vessels of the decidua basalis. These are distinctly different to the narrower, more frequently linked anti-mesometrial vessels shown in (C). (B) but not (C) contains high numbers of CD31+ leukocytes. Image (D) shows the enrichment of leukocytes in decidua basalis while image (E) shows that leukocytes present anti-mesometrially are less frequent and more heterogeneous in shape and size. Red and green are merged in images (F) and (G). Image (F) shows that many cells with co-expression of CD31+ and CD45+ are present mesometrially while image (G) shows their absence anti-mesometrially. Magnification bar in (A) is 200 µm; in B-G 50 µm.

Whole mount immunohistochemistry was used to demonstrate that vessels in early M decidua differ from vessels in AM decidua. These differences are illustrated in Fig. 1 for gd7.5 B6 mice. Even at low power (Fig. 1A), the greater density of CD31+ vessels in the M versus AM or lateral decidua is visible. At higher power, vessels of the M region are seen to be broader and more web-like than in the AM region (compare Fig. 1B to 1C). The webbed appearance is representative of a plexus of immature vessels before pruning and lumen formation in other tissues and in tumors. Under CD31 imaging, the vessels in the secondary AM decidua give the appearance of small, honeycomb-like structures (Fig. 1C), in comparison to the larger distances between related vessels in M decidua (Fig. 1B).

CD45+ cells are present in both M and AM decidua but they are more numerous and more uniformly of lymphoid appearance mesometrially (compare Fig. 1D to 1E). Irregularly shaped CD45+ cells are present throughout decidua but are more noticeable in lateral and AM decidua due to lower CD45+ cell numbers in these regions. The irregularly shaped CD45+ cells were previously classified as CD11b+CD11c-F4/80-Gr-1-CD3-NK1.1-CD31- immature monocytes [8]. When image colors are merged, it is evident that CD45+CD31+ cells are common in M decidua and rare in AM decidua (compare Fig. 1F to 1G). These data illustrate that in live M decidua, leukocytes are abundant and co-localized with vessels in a manner supportive of the hypothesis that they promote angiogenesis. These data also suggest that mechanisms supporting angiogenesis in M decidua differ from those in AM decidua.

### *Dll1* Expression in Mouse Uterine Mucosa and Early Decidua

**Figure 2:**
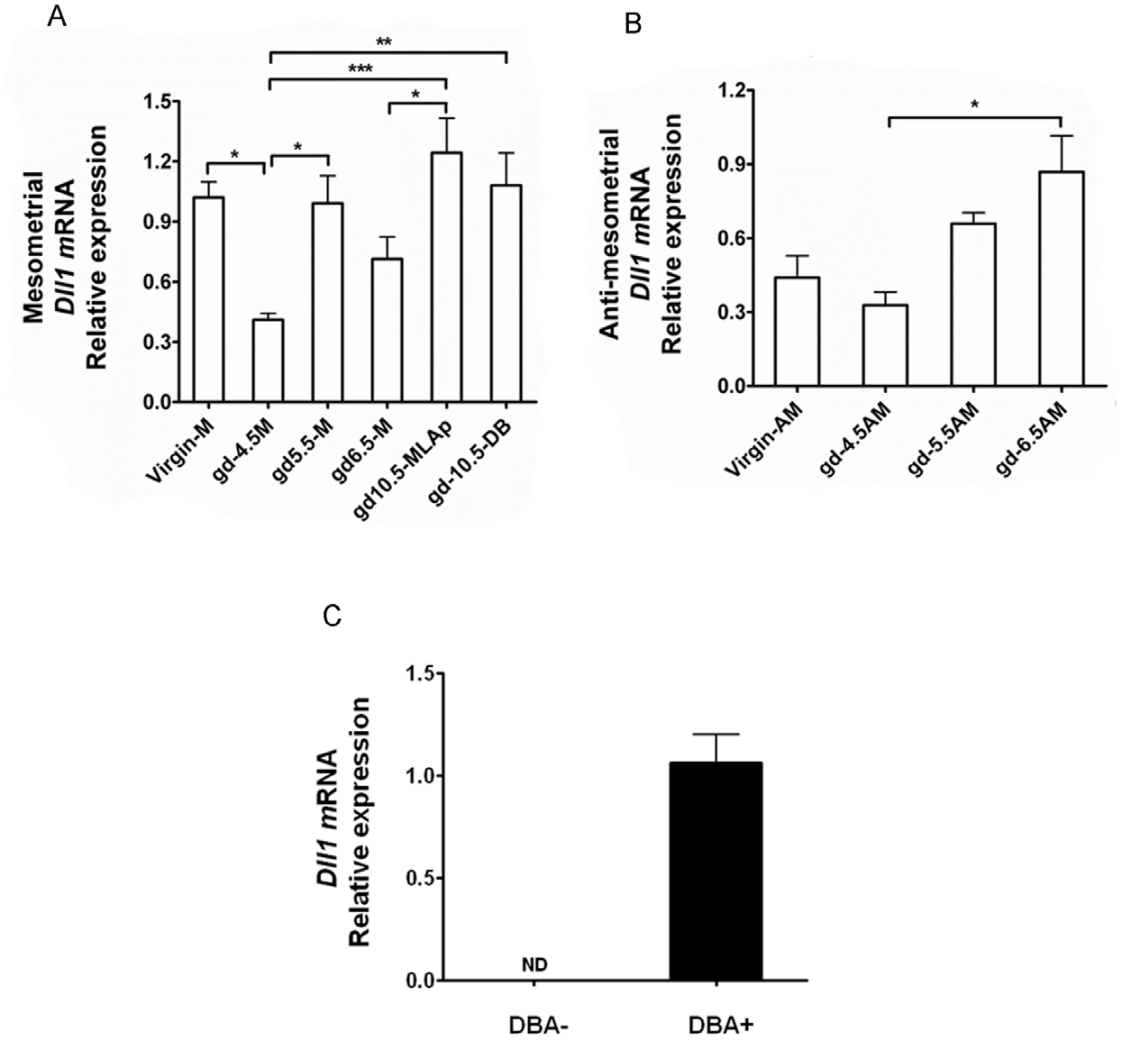
Quantitative realtime PCR analyses of Dll1 in mesometrial and anti-mesometrial uterine samples and in flow sorted uNK cells. Relative mRNA expression of *Dll1* by the mesometrial side (M) of the virgin, gd4.5, gd5.5 and gd6.5 uterus of of the gd10.5 mesometrial lymphoid aggregate of pregnancy (MLAp) and decidua basalis (DB) is shown in (A). Relative mRNA expression of *Dll1* by anti- mesometrial (AM) virgin uterus and by gd4.5, gd5.5 and gd6.5 anti-mesometrial decidua is shown in (B). Relative mRNA expression of *Dll1* at gd10.5 by CD3-CD122+DBA- (DBA-) and CD3-CD122+DBA+ (DBA+) CD1 decidual cells normalized to *Hprt1*(C) is shown in (C). Data are means±SEM from all replicate analyses of two independent experiments. * P<0.05, ** P<0.01, *** P<0.001.

To address *Dll1* expression in the M and AM regions of the virgin uterus, RNA was isolated from diestrous B6 uterine horns that had been transected into M and AM halves. *Dll1* transcripts were detected in both M and AM mucosa (Fig. 2A, 2B). To address whether *Dll1* expression in the uterus was altered by pregnancy, a time course of M and AM *Dll1* expression was conducted using B6 mice. At gd4.5, before decidual angiogenesis is initiated, relative transcript abundance was lower mesometrially than in virgin M uterus. Relative transcript abundance in M decidua then returned to virgin levels at gd5.5 and increased after gd6.5 (Fig. 2A). At gd10.5 when two M regions enriched in uNK cells are present (ie the MLAp and decidua basalis), *Dll1* expression was elevated in each subregion, relative to gd4.5 decidua basalis (Fig. 2A).

In AM tissue, relative abundance of *Dll1* transcripts was similar between virgin and gd4.5 uteri but increased between gd4.5 and 6.5 (Fig. 2B). Studies of AM decidua were not undertaken at gd10.5 due to advanced AM decidual regression at this time. Thus, *Dll1* expressing cells are present in the virgin uterus and in early post-implantation decidua in both M and AM regions. The virgin and AM data indicate that uterine *Dll1* is transcribed by uterine cells other than uNK cells, since classically-characterized uNK cells are absent from these tissues [24].

### *Dll1* Expression in gd10.5 DBA+ and DBA- uNK Cells

To determine whether uNK cells are amongst the M decidual cells expressing *Dll1*, uNK cells were isolated from pooled suspensions of gd10.5 CD1 decidua basalis and MLAp by flow sorting. Transcripts for *Dll1* were detected in RNA from the DBA+ but not the DBA- uNK cells (Fig. 2C). Thus, the uNK cell subset that was previously shown to home to the uterus during pregnancy and to include highly angiogenic uNK cells [26], is the subset that, at gd10.5, contains cells expressing *Dll1*.

### Some DBA+ uNK Cells Express DLL1

**Figure 3:**
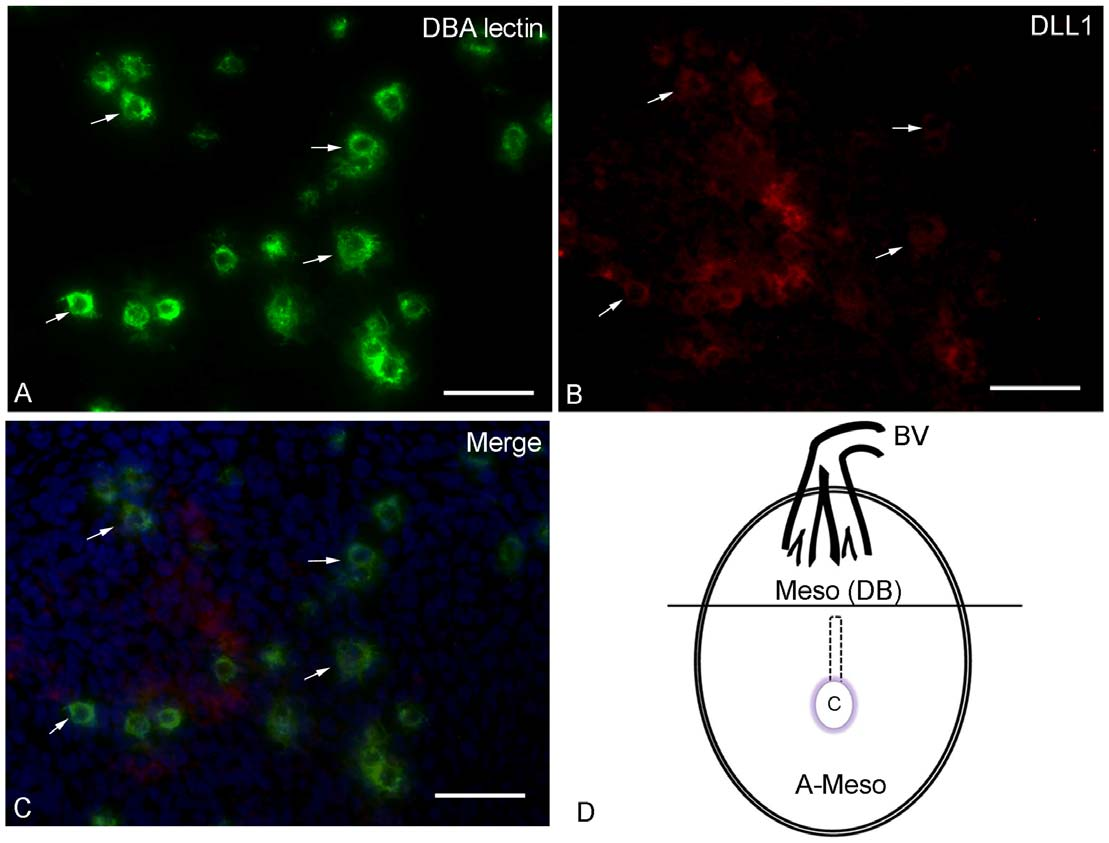
Histological analysis of gd6.5 B6 decidua basalis for expression of DLL1. Photomicrographs of gd6.5 B6 decidua basalis stained with DBA lectin-FITC (green), anti-DLL1-PE (red) and DAPI (blue) demonstrate in (A) DBA lectin-reactive small, agranular uNK cells and immature uNK cells with a few cytoplasmic granules. In (B), the same field is imaged showing cells reactive with DLL1. In the merged image (C), the co-expression of DBA lectin and DLL1 is shown (B and C; arrows mark representative cells). Additional cells that were DBA- and not identified expressed DLL1. The 6.5gd DBA+DLL1+uNK cells were found in the mesometrial decidua basalis (Meso DB) a region indicated as above the horizontal line in drawing (D). BV, entry of major blood vessel branches from the uterine artery; C conceptus, including ectoplacental cone. The area enclosed by dashed lines represents the residual uterine lumen. Bars: A, B and C are 40 µm.

To localize DLL1 expressing cells, whole mount studies were undertaken at gd4.5 and 6.5. Rare, small cells very weakly reactive with DLL1+ were observed anti-mesometrially at both times (data not shown). Because intact, unfixed tissue begins to deteriorate upon an extended antibody incubation that requires agitation, cryostat sections of acetone-fixed gd6.5 and gd10.5 B6 and CD1 implant sites were used for extended overnight antibody incubations to improve the detection of reactive cells. Using PE-anti-DLL1, FITC-DBA lectin and DAPI, DBA+DLL1+ cells were identified in both strains with the only noticable difference between the strains being greater numbers of DBA+ uNK cells in CD1 than in B6. At gd6.5 (Fig. 3A–D), a few cells in decidua basalis were DLL1+ (Fig. 3D). The DLL1+ cells were a mixture of DBA+ (Fig. 3A, 3C) and DBA- (Fig. 3B, 3C) cells. Both the DLL1+ and DBA+ cells had a range of sizes and appeared to be within tissue rather than vessel-associated since DAPI+ nuclei closely abutted cells reactive with either or with both reagents.

**Figure 4:**
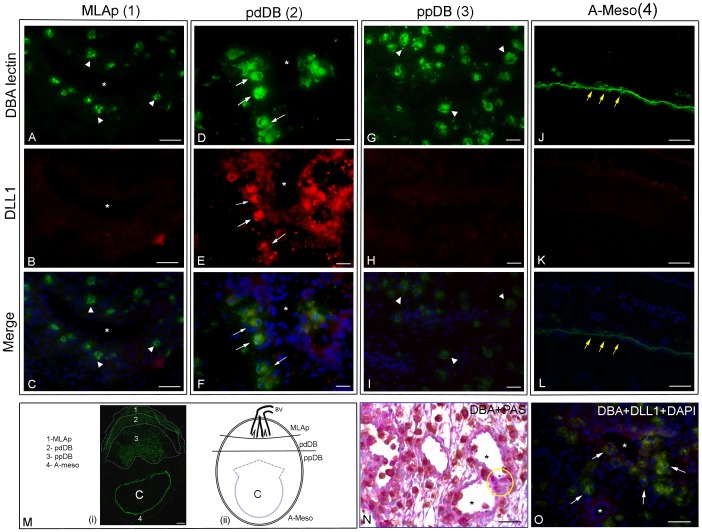
Histological analysis of DLL1 expression in mesometrial sections of gd10.5 B6 and CD1 implant sites. Photomicrographs A-L and O show gd10.5 implant site cryosections co-stained with DBA lectin-FITC (green) to identify uNK cells and anti-DLL1-PE (red). Panel M(i) is stained with only DBA lectin-FITC (green). Panels A–M(i) are B6 implant sites and panel O is a CD1 implant site. Panel M(ii) provides a diagram of the regions images were collected from for the other panels and has been marked to show the banding pattern seen for DLL1 expression. This should be compared to low power image M(i) that shows DBA lectin-stained uNK cell distribution in each banded area. These are labeled region “1” for the MLAp, region 2 for decidua basalis distal to the placenta (pdDB) and region 3 for decidua basalis proximal to the placenta (ppDB). Region 4, the AM decidua, is at the bottom of the image. “C” represents the conceptus, including the placenta and the green ring around “C” is DBA lectin-stained yolk sac endothelium surrounding the fetus. The black space between the yolk sac and the region labeled “3” is the placenta which is uNK cell deficient. Expression of DLL1 by gd10.5 DBA+ cells was stratified within the mesometrial side of the implant sites in both strains and no significant differences were noted. In B6 MLAp (A–C; labeled “1” in M(i) and diagrammed in M(ii)), DLL1 was strongly expressed only infrequently (B) and not by the smaller, immature uNK cells that proliferate in this region (arrow heads in A, C). In decidua basalis of B6 that was distal to the placenta (D–F) and CD1 (O), DBA+ uNK cells brightly expressed DLL1 (arrows in D–F; O). DBA+DLL1+ uNK cells appeared to surrounded vessels (*). Additional perivascular DLL1 staining was present that was not associated with DBA+ cells. The decidual region proximal to the placenta was devoid of DLL1+ cells but abundantly populated by DBA+ uNK cells (G). Neither DLL1+ nor DBA+ uNK cells were present in the highly regressed anti-mesometrial decidua (A-Meso; J-L). DBA-stained yolk sac endothelium was present in this region (arrows in J, L). N is a photomicrograph of the placenta distal decidua basalis in a section from an archived paraffin-embedded gd10.5 B6 implant site double stained using DBA lectin-horseradish peroxidase and Periodic Acid Schiff’s reagent [25]. The latter stain reveals all granulated uNK cells and shows cells of the DBA-PAS+ subset (yellow circle). This image shows the typical strong association of uNK cells with arterioles and with microvessels, including intravascular positions and supports interpretations of the fluorescence images. In M(ii), BV indicates entry of major blood vessel branches from the uterine artery. Bars: A, B, C, J, K, L, O: 40 µm; D, E, F, G, H, I: 20 µm; M: 200 µm.

At gd10.5, cells strongly reactive for DLL1 were readily identified in B6 and in CD1 (Fig. 4A–N show B6; Fig. 4O shows CD1). Importantly, the DLL1+ cells were not uniformly distributed but appeared to be stratified into “layers” or “bands” within decidua basalis (Fig. 4M). In the most external band, “layer 1”, shown in Fig. 4M(i), DLL1+ cells were absent from the myometrium (not shown) and very rare (Fig. 4B) in the MLAp in comparison to DBA+ uNK cells (Fig. 4A). The rare DLL1+ cells in the MLAp were vessel associated (Fig. 4B, 4C). Decidua basalis was subdivided into two layers on the basis of DLL1 expression (labeled 2 and 3 in Fig. 4M(i)). The decidua basalis more distal to the placenta (pdDB) contained numerous large cells with bright expression of DLL1 (Fig. 4E). These cells co-expressed DBA lectin (Fig. 4D, 4F showing B6; Fig. 4O showing CD1), identifying them as DLL1+ uNK cells. The DLL1+DBA+ uNK cells appeared to be aligned around vessels. This is a typical position for DBA+ and DBA- uNK cells in paraffin-embedded sections (Fig. 4N). DBA- uNK cells are identified by PAS staining which reveals all uNK cells with cytoplasmic granules. DBA- cells of undefined lineage(s) with dim DLL1 expression were also present in M decidua of B6 and CD1 (Fig. 4F, 4O) indicating that uNK cells are an abundant but not exclusive source for this ligand. Decidua basalis proximal to the placenta (ppDB) contained the highest frequency of uNK cells (Fig. 4G) but lacked DLL1 expressing cells (Fig. 4H, 4I). Additionally, AM decidua lacked detectable DLL1 expression (Fig. 4K, 4L) and lacked DBA+ uNK cells (Fig. 4J). The only DBA+ cells in AM regions were endothelial cells of the yolk sac (arrows in Fig. 4J, 4K).

## Discussion

Application of whole mount *in situ* immunohistochemistry to mouse uterus between implantation and mid pregnancy (gd9.5) was recently reported [8]. In that study, no direct interactions were observed between decidual CD45+ cells and trophoblast cells that ubiquitously expressed a fluorescent gene tag. That observation made in intact, viable implant sites challenged a number of widely held ideas concerning direct receptor-ligand and cell contact interactions between trophoblasts and the uNK cells recruited to early decidua basalis. In contrast, the whole mount study provided positional information suggesting early decidual CD45+ cells act on the autologous vasculature of the mesometrial decidua. Neoangiogenesis accompanies decidual development and is crucial for normal gestational development [16], [32], [33]. Both human and mouse uNK cells produce the angiogenic molecules VEGF and PGF that regulate endothelial cell division. Time course and uNK cell subset analyses of VEGF expression further showed that between gd7.5–9.5, 50% of DBA lectin+ uNK cells express VEGF. By gd14.5, VEGF+DBA+ uNK cells were 30% of the DBA lectin+ uNK cells and total uNK cell numbers had dropped suggesting the angiogenic roles of uNK cells regress after mid gestation [28]. Recently, microarray analyses and validations were reported that reached the conclusion that mouse uNK cells do not contribute to decidualization and angiogenesis [34]. In that study, decidua from CD1 mice with and without *Il15,* the gene for an essential growth factor in uNK cell differentiation, were compared at gd7.5. Other studies, including ultrastructural studies, of uNK cell deficient mice [35], conducted between gd 6.5 to 14.5 [28], [35]–[37], suggest that gd7.5 was at least one day too early to observe effects from absence of uNK cells on decidual cell numbers or decidual vessels. DLL1 has a critical, non-mitogenic role in neoangiogenesis because it triggers the induction of tip cells, in a cell contact-dependent process that is central to the initiation of arterial branching angiogenesis. This allows proliferation in cells next to the differentiated tip cell, the stalk cells, to extend the vessel. The direction of new growth is determined by factors that influence the tip cells [38]. Studies of neonatal mouse retinal vascular development indicate that DLL1 is secreted by non-endothelial cells and leads to orthogonal/perpendicular vascular growth. We found DLL1 expressing cells at a very low frequency in mouse decidua at gd4.5 by whole mount staining as might be expected prior to onset of angiogenesis. Given the report of significant decidual angiogenesis including sprouting angiogenesis at gd6.5 [8], an unexpectedly small increase in DLL1+ cells was seen in whole mounts at gd6.5 and again, only in AM decidua. One explanation of these results could be that the AM angiogenesis was not arterial since DLL1 is strongly associated with arterial differentiation [39]. An alternate explanation could be that the 1 h incubation used in whole mount staining to maintain tissue viability was insufficient for strong binding of this antibody. For extension of the antibody incubation time to an overnight staining protocol, cryostat sections of fixed implant sites rather than viable hemisections were used. Cryostat sections were prepared from both B6 mice in which we had molecular evidence for *Dll1* expression in M decidua and from CD1 mice in which we had evidence for *Dll1* expression in uNK cells. With this approach, DLL1 expression was detected at gd6.5 in DBA+ uNK cells in decidua basalis. The data of Paffaro et al., [24] suggest that >90% of the DBA+ uNK cells present in M decidua at gd6.5 (7.5 in the counting of their study), are immature. The remaining DBA+ uNK cells are larger, more mature cells containing numerous cytoplasmic granulates that are also DBA-reactive. Both smaller, non-granulated and larger, lightly granulated DBA+ uNK cells expressed DLL1 (Fig. 3) suggesting DLL1 expression is a hallmark of this lineage subset and not a protein acquired late during uNK cell maturation. The absence of significant DLL1 staining at gd10.5 in similar early stages of uNK cells found in the MLAp (Fig. 4 A–C) shows that DLL1 expression is dynamic during pregnancy and suggests influences from the stromal micro-environment or gestational length.

Previous comparative studies on cultured NK cells isolated from first trimester human termination decidua and from blood showed that uNK cells but not blood NK cells elevate production of interferon gamma (IFNG) but not of interleukin 8 (IL8) in response to exogenous DLL1 [22]. When combined with our data, this finding potentially suggests that autocrine DLL1 responses may occur in mouse uNK cells, as in endothelial cells [2]. Analysis of IFNG was important since its production by uNK cells initiates spiral arterial remodeling at mid pregnancy [40]. However, IFNG regulation in mouse uNK cells cannot be achieved by autocrine regulation since DBA- uNK cells that lack DLL1 expression are the mouse IFNG-producing uNK cell subset [26]. From studies of human hematopoietic stem cell cultures, it was found that exogenous DLL1, DLL4 or Jagged2 but not DLL3 or Jagged1 promoted differentiation of NK cells with the decidual CD56+CD16- phenotype [41]. Thus, the most probable interpretation of our data would be that angiogenic, DBA+ uNK cells expressing DLL1+ and having autocrine capacity act on DBA-DLL1- uNK cells that express Notch receptors to elevate IFNG production [26], [42]. Peak IFNG production in mouse decidua basalis is at gd10.5–12.5 [43], consistent with the transient high expression of DLL1 in DBA+ uNK cells at gd10.5.

NK cells are now grouped under the umbrella of innate lymphoid cells (ILC). This cell category, important in mucosal tissues, includes lymphoid tissue inducer (LTi), NK22 and nuocytes or ILC2 cells [44]. Exactly how uNK cells relate to these various lineages is at present unclear. LTi contribute to the development of lymph nodes and intestinal lymphoid structures including Peyer’s Patches and are characterized by their cytokine profile. UNK cells, like LTi cells, express IL22 [26] and IL7RA [45] and are associated with development of a lymphocyte-enriched region. Our finding that DLL1 is a product of immature and mature uNK cells suggests it would be profitable to explore the roles of other ILC subsets in the promotion of angiogenesis and in particular in the induction of endothelial tip cell differentiation. Early angiogenic actions could be major roles of ILCs essential in the promotion of secondary lymphoid tissue development.
